# The role of information and communication technology on the well-being of residents in rural nursing homes

**DOI:** 10.1093/heapro/daaf194

**Published:** 2025-11-26

**Authors:** Xiaoli Li, Craig McPherson, Manasa Battu, Thomas Shaw

**Affiliations:** Southern Illinois University Carbondale, School of Health Sciences, 1365 Douglas Drive, Carbondale, IL 62901-6604, United States; Southern Illinois University Carbondale, School of Health Sciences, 1365 Douglas Drive, Carbondale, IL 62901-6604, United States; Southern Illinois University Carbondale, School of Health Sciences, 1365 Douglas Drive, Carbondale, IL 62901-6604, United States; Southern Illinois University Carbondale, School of Health Sciences, 1365 Douglas Drive, Carbondale, IL 62901-6604, United States

**Keywords:** information and communication technologies, residents’ well-being, rural nursing homes, ICT benefits, ICT support

## Abstract

Information and communication technologies (ICTs) are increasingly recognized as valuable tools for enhancing the well-being of nursing home residents. However, residents’ perspectives on ICT usage and its implementation in rural nursing homes remain underexplored. This study investigated the role of ICT in promoting well-being from the viewpoint of residents in rural long-term care settings. A qualitative research approach was employed, using semistructured interviews with 16 residents from nursing homes in southern Illinois, USA. Thematic analysis generated five key themes: ICT as a bridge to the outside world, ICT as mental and emotional support, ICT’s role in enhancing independence and self-sufficiency, Technology remains out of reach, and ICT support and assistance. The findings highlight the significant advantages of ICT use, particularly in improving mood, reducing isolation, and alleviating symptoms of depression. Despite these benefits, ongoing barriers, such as limited resources, financial constraints, and insufficient institutional support, continue to impede effective ICT integration. These challenges underscore the need for increased investment in digital literacy programs, reliable internet access, and affordable technology solutions to maximize the benefits of ICT in rural long-term care settings.

Contribution to Health Promotion

- The study demonstrates how information and communication technology (ICT) use in rural nursing homes can reduce isolation, alleviate depression, and improve overall mood among residents.
- By highlighting residents’ use of ICT for communication, entertainment, and daily activities, the research supports the role of technology in fostering autonomy, social connectedness, and cognitive engagement.
- The study uncovers systemic challenges to ICT adoption, such as financial and infrastructural limitations, providing actionable insights for designing health promotion strategies that target digital inclusion and equity in long-term care settings.

## BACKGROUND

The US Census Bureau estimates that adults aged 65 and older will constitute 20% of the population by 2030, with the percentage of those aged 85 and older projected to increase from 1.9% in 2012 to 4.5% by 2050 (Ortman *et al*. 2014). As the older adult population grows, nursing homes have become essential in providing medical, social, and supportive care to individuals who can no longer live independently due to chronic illness, cognitive decline, or physical impairments (Doty *et al*. 1985, Heiks and Sabine 2022). However, nursing home residents often face significant challenges, including loneliness and depression, which profoundly impact their health and quality of life. Studies indicate that up to 50% of older adults in long-term care facilities experience significant loneliness (Perissinotto *et al*. 2012), while 30%–40% suffer from clinically significant depression (CDC 2024). These issues are exacerbated by social isolation, loss of independence, and limited access to mental health services, all of which contribute to depressive symptoms (Cohen-Mansfield and Perach 2015). The consequences of loneliness and depression are far-reaching, with research linking them to increased risks of cognitive decline, cardiovascular diseases, and mortality (Cacioppo *et al*. 2015, Holt-Lunstad *et al*. 2015). Moreover, these conditions reduce residents’ ability to engage in social activities, lower life satisfaction, and hinder overall well-being (Djernes 2006).

Despite growing awareness of these challenges, addressing loneliness and depression in nursing homes remains difficult. Traditional interventions, such as group therapy or one-on-one counseling, have been shown to reduce depressive symptoms and feelings of isolation when implemented appropriately (Cattan *et al*. 2005, Dickens *et al*. 2011). However, their effectiveness is often moderate and highly dependent on consistent participation, quality of facilitation, and residents’ willingness to engage. Attendance and engagement can be limited by mobility or health constraints, and the stigma surrounding mental health may prevent residents from seeking help (De Mendonça Lima and Ivbijaro 2013, Li *et al*. 2024). Additionally, these interventions are frequently resource intensive, requiring trained personnel, dedicated spaces, and ongoing organizational support, resources that many facilities lack (Hoang *et al*. 2022).

Given these limitations, there is a pressing need to explore technological, nonpharmacologic approaches to improve emotional well-being and cognitive function among nursing home residents. While digital interventions hold potential, they also require trained personnel and infrastructure for implementation and should be considered complementary strategies that help address the practical and social barriers faced by traditional interventions (Alotaibi *et al*. 2025).

### Information and communication technology

Information and communication technologies (ICTs) encompass devices and applications that facilitate access to information and enable electronic communication, such as sending text messages or participating in video calls. Common examples include mobile phones, smartphones, computers, tablets, laptops, and internet connectivity (Schlomann *et al*. 2020). In the broader aging literature, ICT has been shown to transform everyday life by enabling new forms of social participation, interaction, and enhanced access to information among community-dwelling older adults (Antonucci *et al*. 2017, Kim *et al*. 2017, Castellacci and Tveito 2018). Older adults who do not use ICT devices risk being socially excluded from an increasingly digital society (Cotten 2017, Olsson *et al*. 2017). Evidence linking ICT use with well-being is mixed: some studies in community settings show reduced loneliness (Lelkes 2013, Szabo *et al*. 2019), whereas others, such as a study of computer and internet use, have not demonstrated significant benefits (Castellacci and Tveito 2018). In contrast, research in long-term care settings highlights the distinct potential of ICT to address social isolation and limited opportunities for engagement. In nursing homes and similar facilities, ICT has been shown to alleviate loneliness, enhance cognitive stimulation, and improve quality of life (Cotten *et al*. 2013). For example, video calling applications and structured ICT programs provide residents with vital connections to family and friends, compensating for restricted mobility and limited access to external social networks (Tsai and Tsai 2010, Tsai *et al*. 2020).

These findings highlight the promise of ICT as a tool to support well-being in long care environments. Yet, questions remain about how ICT is taken up and experienced in rural nursing homes, where limited resources and support may shape its role in residents’ daily lives.

### Rural nursing homes

While urban nursing homes are often located within easy access to digital infrastructure and healthcare services, rural nursing home communities face unique structural and logistical challenges. The term “rural” varies in definition across classification systems but generally refers to areas with less concentrated populations and greater distances from urban centers. The US Census Bureau and the Office of Management and Budget (OMB) define rural areas as nonmetropolitan counties, which typically lack city-level healthcare and technology amenities (Cromartie and Ratcliffe 2025).

Residents of rural nursing homes are particularly affected by geographical isolation, shortages of healthcare personnel, and limited access to broadband infrastructure, all of which hinder the effective use of ICT. Importantly, most residents in rural nursing homes tend to come from nearby communities, meaning their digital skills and experiences are shaped by the broader rural digital context prior to admission. This is significant given the well-documented urban–rural digital divide in older populations: older adults in rural areas are less likely to use the internet and engage with digital media compared to their urban counterparts (Choi *et al*. 2021, Rosenberg and Nimrod 2021, Crowe *et al*. 2024). Compounding these issues, many nursing home residents face individual barriers such as technological illiteracy, sensory deficits, and cognitive impairments, which further limit their ability to use digital technologies effectively (Boamah *et al*. 2021a, 2021b, Healthcare Access in Rural 2024).

Together, these constraints highlight the importance of designing sustainable ICT solutions that not only provide infrastructure but also account for residents’ prior digital histories and the specific needs of rural nursing home communities.

### Theoretical foundations

This research explores the role of ICT in rural nursing homes within the framework of environmental gerontology. Environmental gerontology is a theoretical approach that examines the relationship between older adults and their physical and social environments. A key concept in this framework is Person–Environment Fit (P–E Fit), which focuses on the interaction between an individual’s competencies (e.g. physical, cognitive, and emotional abilities) and the demands or supports provided by their environment (Lawton 1982). In the context of ICT use, this framework explains how technology can act as both a support and a barrier for older adults, depending on its usability and the individual’s digital literacy. Older adults are active agents who seek to modify or adapt to their environments through strategies such as learning new technologies, using digital tools to maintain social connections, or accessing health information online (Gitlin 2003). With the growing integration of ICT in care settings, environmental gerontology has evolved to encompass person–technology fit, recognizing that technology is now a crucial component of the modern environment (Czaja and Sharit 2009). Thus, ICT adoption in later life can be understood as a process of adaptation and self-regulation, in which individuals balance their changing capacities with the increasing digital demands of daily living (Forsman *et al*. 2018, Wahl and Gerstorf 2020).

We expect a more beneficial P–E Fit when individuals use modern technologies, as this indicates successful adaptation to the changing technological context. According to the principles of environmental gerontology, a good fit between the person and their environment leads to greater independence, well-being, and quality of life for older adults (Lawton 1982). In this regard, ICT can serve as an enabling resource that enhances resilience, supports social participation, and promotes overall quality of life in later years.

### The present study

While ICT is increasingly recognized as a tool to enhance well-being among nursing home residents, its implementation in rural nursing homes remains underexplored. Existing research predominantly focuses on urban healthcare settings, which typically benefit from high digital proficiency, broadband penetration, and financial resources, making technology more accessible. In contrast, rural nursing home settings face systemic challenges such as inadequate infrastructure, limited funding, and low levels of digitalization (Henning-Smith *et al*. 2021, McKnight’s Senior Living 2023).

Furthermore, qualitative research evaluating resident attitudes toward ICT adoption in rural long-term care homes, as well as its effects on daily life, social connectedness, and cognitive health, has been scarce. This study aims to address this research gap by exploring the following research questions using qualitative methodology:

- In what ways do rural nursing home residents interact with and make use of technologies such as smartphones, tablets, and video calling applications?
- How does the use of ICT influence the emotional well-being, social connectedness, and cognitive engagement of residents in rural nursing homes?
- What experiences and perceptions do residents in rural nursing homes have regarding the barriers and support of ICT use?

## METHODOLOGY

### Study design

This study employed a qualitative study design as outlined by Baxter and Jack (2008), utilizing in-depth, semistructured interviews to gather insights into the impact of ICT on residents’ quality of life. Semistructured interviews were chosen because they provide both consistency and flexibility, allowing participants to share rich, personal experiences while enabling the researcher to probe for deeper insights, making them especially suitable for exploring sensitive issues among older adults.

### Participants and settings

Older adults living in two nursing homes located in rural southern Illinois, USA, participated in this study. These facilities were selected because they provide a representative sample of long-term care settings that serve older adults with high levels of dependency and limited access to resources. Both facilities offer skilled nursing, rehabilitation, and long-term care services. Their annual occupancy rates range from 55% to 59%, and most residents require 24/7 care and rely primarily on Medicare and Medicaid to cover service costs (Healthcare Management Partners Metrics 2025, Pritzker 2025).

The final sample comprised 16 older adults (women, seven; men, nine; mean age = 67 years). To be eligible for interview, they needed to be residing at a nursing home for at least 3 months, self-identify an ability to respond to questions, and be willing to share their experiences (see Table 1). No respondents were excluded for technical or logistical reasons outside of the predefined inclusion criteria. All individuals who met the criteria and expressed willingness to participate were successfully interviewed.

**Table 1.** Demographics of residents (*N* = 16).

Variables	Characteristics	*n*	%
Age	<60	2	12.50%
	60–64	1	6.25%
	65–69	1	6.25%
	70–74	3	18.75%
	75–79	4	25%
	80–84	3	18.75%
	85	2	12.50%
Gender	Male	9	56.25%
	Female	7	43.75%
Marital status	Single	4	25%
	Married	7	43.75%
	Widow	2	12.50%
	Divorced	3	18.75%
Education	High school	7	43.75%
	Some college	4	25%
	Associate degree	2	12.50%
	Bachelor’s degree	1	6.25%
	Unknown	2	12.50%
Number of outside visitors	None	2	12.50%
	Not often	5	31.25
	<3 times per week	4	25%
	>5 times per week	2	12.50%
	<3 times per month	2	12.50%
	Unknown	1	6.25%
Number of weekly outside activities	None	2	12.50%
	Not often	3	18.75%
	Medical visit	5	31.25%
	<2 times per week	3	18.75%
	1 per month	1	6.25%
	Unknown	2	12.50%

### Data collection

Participants began by self-reporting their demographic information, including age, gender, and marital status. Following this, they took part in semistructured interviews. Sample interview questions included the following: “What electronic devices do you use now? For what purposes?”. “What are the advantages of using ICT to your wellness?”, “What is most challenging for you when using ICT? Why or why not?”, and “What kind of support or help you need when you use the ICT?”. Participants responded to a total of 10 core interview questions along with follow-up probes (see Table 2), allowing us to gather detailed insights into their experiences with ICT.

**Table 2.** ICT interview questions with follow-up probes.

Interview questions	Follow-up probes
1. What electronic devices do you use now?	Which device do you use most and why? Any difficulties accessing them?
2. What apps/programs do you use? For what?	How did you learn to use them? Any apps you wish you could use?
3. What are the advantages of using ICT for your wellness?	Can you give examples of how it improves mood, social connection, or daily activities?
4. What is most challenging for you when using ICT?	Can you describe a frustrating experience? How does it affect your use of ICT?
5. What kind of support or help do you need when using ICT?	Who helps you and how often? What would make ICT easier to use?
6. How do you feel when using an electronic application?	Confident, anxious, or neutral? Any recent positive or negative experiences?
7. What is your attitude toward using ICT?	Has it changed over time? What influences your attitude?
8. What role does ICT play in your daily life?	How much time do you spend using it? Does it affect routines or social interactions?
9. How does your community support you in using ICT?	Are staff helpful? Are there group programs for learning ICT?
10. What improvements would you like to see in technology at the facility?	Specific devices, apps, or services desired? How would they impact daily life?

### Procedure

The study was approved by the Institutional Review Board (IRB) of Southern Illinois University Carbondale and by the managers of the participating nursing homes. Informed consent was obtained from all residents prior to their participation. The residents who agreed to participate engaged in an in-person interview, which allowed for more in-depth discussions about their experiences with ICT. Each interview took ∼20–30 minutes and was audio-recorded. Interviewers also took detailed notes during the sessions. Interviews were transcribed manually, and all personal identifying information was removed, with each participant assigned a pseudonym for confidentiality.

### Analytic strategy

Qualitative data were analyzed using thematic analysis following Braun and Clarke’s six-step approach (2019, 2022): (i) familiarization with the data, (ii) generating initial codes, (iii) developing themes, (iv) reviewing themes, (v) refining and naming themes, and (vi) producing the report. After transcription, the research team immersed themselves in the data to identify meaningful segments, and codes were generated iteratively, informed by the research questions and participants’ context. These codes were then organized into preliminary themes, which were reviewed and refined across the entire dataset to capture patterns spanning multiple questions.

The research team actively engaged in reflexive practice throughout analysis. All four authors independently coded portions of the data, followed by team discussions to examine interpretations, challenge assumptions, and ensure consistency in theme development. Positionality was considered, acknowledging how the researchers’ experiences and perspectives may have influenced coding decisions and the selection of illustrative excerpts. Each participant was assigned a unique identifier (P1–P16), and member checks were conducted to enhance credibility. All data were managed in Word, and coding and theme development were documented in an iterative, reflective process, ensuring rigor and transparency in line with thematic analysis principles.

## FINDINGS

Participants’ experiences in using ICT were discussed, starting from their general attitude to ICT usage, the purpose of using ICT, and the benefits, barriers, and recommendations (see Table 3). Through inductive thematic analysis, we developed five key themes: ICT as a bridge to the outside world, ICT as mental and emotional support, ICT’s role in enhancing independence and self-sufficiency, Technology remains out of reach, ICT support and assistance (see Table 3)

**Table 3.** Analysis themes.

Themes	Subthemes
1. ICT as a bridge to the outside world	Communication and social networking
	Entertainment and information access
	Online shopping and convenience
2. ICT as mental and emotional support	Engagement and cognitive stimulation
	Emotional well-being
3. ICT’s role in enhancing independence and self-sufficiency	Emotional and social dependence on ICT
	Digital independence and self-sufficiency
4. Technology remains out of reach	Limited accessibility
	Financial constraints
	Technical and usability challenges
	Health-related barriers
5. ICT support and assistance	Access to reliable internet and digital resources
	Digital literacy and ICT support services

### Theme 1: ICT as a bridge to the outside world

Participants reported using a variety of ICT tools, most commonly smartphones and tablets, followed by video calling applications such as FaceTime and social media platforms such as Facebook. Frequency of use varied: several residents described daily engagement for communication, entertainment, or information access, while others used ICT only a few times per week, often depending on internet reliability, device availability, or physical health. A small number of participants reported minimal or no use, typically due to lack of confidence or interest.

This theme explored the diverse ways nursing home residents engaged with ICT for communication, entertainment, and convenience. The findings revealed that access to digital tools contribute not only to maintaining social ties but also to fostering emotional well-being.

### Communication and social networking

Residents use ICT to maintain relationships with family and friends, helping reduce feelings of loneliness and social isolation.

“I currently have a cellphone, a laptop computer, and a TV. I use Facebook to stay connected with my family members and friends” (P6)

“Facebook was helpful in finding my grandson, although I have not been in touch with him recently.” (P5)

“I have a tablet and a smart TV. I primarily watch Netflix, especially on weekends. I play interactive games and have made good friends through them on smartphone. I use LINE, a messaging app, for calls, messages, and group chats.” (P3)

### Entertainment and information access

ICT tools provided residents with entertainment options and up-to-date information, contributing to cognitive engagement and emotional satisfaction.

“I watch YouTube, I have Facebook, just look and see what other people have sent me—that’s how I keep track of them. I have a coloring book that I color all the time on my phone.” (P17)

“Yahoo is my go-to platform for reading news and staying updated on current events.” (P6)

“If I’m sick, I look up what’s wrong with me and see if I can find out how to handle it. I also use Google to look up health-related information.” (P8)

### Online shopping and convenience

Residents expressed appreciation for the convenience of using online platforms to purchase personal items independently.

“I have also used online platforms to buy various items, which makes shopping more convenient.” (P6)

“I also used online platforms like Amazon and Walmart to purchase items, which was convenient.” (P17)

These statements reflect the value of ICT in providing connections to family and friends, which assists in relieving feelings of isolation or loneliness. The statements also indicate enhanced autonomy through the ability to purchase personal items without reliance on facility staff assistance. Participants actively using ICT described it as an essential part of their daily routines, providing both practical utility and personal satisfaction. Through the lens of P–E Fit, residents’ competence in using digital technologies matched opportunities provided by their environment, allowing them to engage meaningfully with ICT. Environmental gerontology emphasizes that aging individuals thrive when their abilities align with environmental demands and resources. Participants who had access to user-friendly devices and familiar software experienced increased autonomy.

### Theme 2: ICT as mental and emotional support

This theme highlighted the perceived advantages of ICT use among nursing home residents, particularly in fostering cognitive engagement, maintaining social connections, and supporting autonomy in everyday life.

### Engagement and cognitive stimulation

Residents expressed that ICT use offered valuable mental exercise and alleviated feelings of boredom or isolation.

“It is a good way to practice, to exercise, to concentrate on something, and to train your brain to react to it. Takes away a lot of boredom here.” (P6)

“Music is a big part of my life as I am a musician. I play the piano, and ICT allows me to enjoy and explore music online.” (P4)

### Emotional well-being

Technology facilitated ongoing communication with loved ones and nurtured a sense of belonging, providing emotional support.

“Technology allows me to stay connected with people and participate in meetings remotely via Zoom and Skype. This is crucial, as it prevents isolation and helps me maintain friendships. Without technology, I would feel much more disconnected from the outside world.” (P15)

“It helps keep my mind busy and allows me to communicate with my sister and other friends. ICT helps me stay engaged in different activities.” (P3)

“It provides a sense of connection that I would not have otherwise.” (P7)

ICT supported residents’ emotional well-being and cognitive stimulation, reflecting a positive P–E Fit where technology served as a mediator between personal needs and environmental opportunities. Participants reported feeling emotionally stable and cognitively engaged when they could interact digitally with family, friends, or the broader world, which enhances the fitness between residents’ needs and the social resources accessible within and outside the nursing home. When devices and connectivity were available, residents maintained relationships and reduced feelings of isolation, supporting overall well-being.

### Theme 3: ICT’s role in enhancing independence and self-sufficiency

Participants who actively use ICT expressed a strong appreciation for its role in enhancing their daily experiences. ICT is not only viewed as a tool for convenience but also as a vital resource that supports emotional well-being and personal independence.

### Emotional and social dependence on ICT

Several participants shared how essential digital devices have become to their emotional stability and ability to maintain close relationships. For many, ICT served as a lifeline to the outside world.

“I wouldn’t survive without my phone. I must have it. That’s my connection to the world… I couldn’t access anything, felt irritated.” (P16)

“I couldn’t imagine not having it in my life at this time… Being away from family, I couldn’t imagine not having it.” (P3)

“I rely on it to stay in touch with my wife. I would feel lost without it.” (P7)

### Digital independence and self-sufficiency

Participants also described how ICT use fostered a sense of autonomy. Rather than depending on others, some residents felt empowered by their ability to access information and assist others.

“I enjoy using technology to learn and find information. People often ask me questions, and I like looking things up.” (P4)

“I don’t need much help; I’m usually the one assisting others with ICT.” (P3)

“ICT has helped me book medical appointments online and stay updated with my prescriptions.” (P6)

Residents highlighted how ICT fostered autonomy, aligning personal capabilities with environmental affordances. Using P–E Fit, this theme reflects situations where the environment supports residents’ desire for self-sufficiency, such as accessing information, managing appointments, or assisting peers. Where the environment matched residents’ abilities (e.g. available devices and functional interfaces), participants reported enhanced control and confidence.

### Theme 4: technology remains out of reach

While many participants recognized the benefits of ICT, they faced a range of barriers that limited full utilization. From a P–E Fit perspective, these challenges reflect a misalignment between residents’ personal capabilities and the environmental demands or support necessary to use technology effectively. Environmental gerontology emphasizes that aging outcomes are shaped not only by individual abilities but also by how well the environment accommodates those abilities. In this theme, the nursing home environment often failed to provide the resources and adaptations needed for optimal ICT engagement.

### Limited accessibility

Residents reported inconsistent or unavailable internet connectivity, highlighting a structural misfit between personal needs for digital engagement and the facility’s technological environment.

“I can’t get internet on here. I can’t access it on the tablet or anything.” (P4)

“The facility provides Wi-Fi, but it is currently turned off, which limits my ability to use the internet freely.” (P6)

### Financial constraints

The cost of owning and maintaining devices was a recurring concern among participants.

“Currently, I do not own a smartphone due to financial constraints.” (P5)

“I don’t have a phone or computer, partly because of the cost and getting it for me. It’s not challenging for me to use it; I just don’t have one.” (P1)

### Technical and usability challenges

Residents frequently express confusion, frustration, and insecurity when navigating ICT, indicating a mismatch between individual competence and technological complexity.

“I think it’s difficult. If you try something, your computer can get all out of whack… I can’t remember my password. I have a cellphone, and I can’t use it. I screw it up every time I try.” (P8)

“I find ICT confusing. There are too many settings and features that make it overwhelming for me.” (P5)

“Smartphones and computers are hard to use.” (P14)

### Health-related barriers

Cognitive and physical health limitations are further complicated ICT use. Memory impairments, visual difficulties, and reduced concentration created a misalignment with the environmental demands of digital tools.

“I had a stroke too, so it’s hard for me to concentrate and stay on something for a long time.” (P8)

“One of the biggest challenges I face is remembering passwords and phone numbers… I need glasses to see the screen properly, but I keep breaking them… I tend to forget instructions due to short-term memory issues.” (P5)

Across accessibility, financial, technical, and health-related domains, the findings reveal persistent misfits between residents’ capabilities and environmental resources. Nursing home settings often lacked the infrastructure, affordability, and support systems necessary to optimize ICT use. Applying a P–E Fit framework highlights that ICT engagement is not solely dependent on individual motivation or competence but is critically shaped by how well the environment accommodates residents’ needs. Optimizing this fit through reliable connectivity, financial support, usability adaptations, and targeted training could significantly enhance residents’ digital inclusion and overall quality of life.

### Theme 5: ICT support and assistance

The availability and quality of institutional support significantly influenced the extent to which nursing home residents can benefit from ICT. Participants described both physical access issues and a lack of digital literacy support from health providers, highlighting the need for a more structured and proactive approach from facility leadership.

### Access to reliable internet and digital resources

Several participants expressed frustration with poor internet connectivity and insufficient technical infrastructure, which directly limited their ability to use personal devices.

“I haven’t been able to find anybody yet, but they’re not really all that cooperative about helping you with your stuff like setting up the Wi-Fi… I would like to have better internet access. The facility does not provide reliable Wi-Fi, which prevents me from using my tablets effectively.” (P16)

“The facility’s Wi-Fi can be unreliable, but I use my phone’s data as a backup.” (P15)

### Digital literacy and ICT support services

Residents also noted the absence of structured training opportunities and technical assistance, which are essential for older adults to feel confident in using technology.

“Having an area where residents can sit and get on the computer themselves and do what they need to do or having a device they can carry to their rooms would be helpful. Training in technology as well.” (P10)

“I’ve complained that they don’t know what to do about it. So apparently, they’re not too electronically literate around here. Yeah.” (P16)

Participant responses indicate that institutional support and technical assistance significantly shaped residents’ ability to benefit from ICT. P–E Fit emphasizes the alignment between residents’ competencies and the environmental supports provided. When support was lacking, misalignment between resident abilities and environmental affordances led to frustration and decreased engagement.

Across all five themes, P–E Fit provided a conceptual lens to understand how older adults interact with ICT in nursing home settings. Environmental gerontology emphasizes that successful aging depends not only on individual capacities but also on the adaptation of physical, social, and technological environments.

## DISCUSSION

Our findings revealed that the use of ICT among older adults living in nursing homes can significantly enhance emotional well-being, social engagement, and cognitive stimulation. However, the findings also indicated that serious barriers to ICT adoption and utilization remain—such as limited resources, inadequate infrastructure, and low digital literacy. This research further explored the unique role of ICT in rural nursing homes, where access to healthcare, social services, and in-person visits was often limited.

### ICT enhances mood, communication, and engagement

Specifically, our study reveals that ICT has a positive emotional impact by increasing interaction with family, alleviating loneliness, and supporting engagement in meaningful activities. The findings align with previous literature demonstrating that ICT use among older adults reduces loneliness, enhances social participation, and stimulates cognitive engagement (Cotten *et al*. 2013, Ge *et al*. 2018).

Beyond communication, ICT also serves as a valuable source of entertainment and mental stimulation for residents in long-term care settings. Residents often use platforms such as YouTube to access music or engage in digital games and creative design programs, allowing them to maintain personal interests, pursue hobbies, and express creativity. This individualized, interest-driven use of technology supports cognitive engagement, fosters a sense of purpose, and promotes emotional well-being. These observations align with previous research indicating that active ICT use among older adults can enhance cognitive function, increase autonomy, and improve emotional outcomes (Czaja and Sharit 2009, Charness and Boot 2016). The integration of technology for leisure and mental activity highlights its broader role as a component of holistic care in long-term care environments. Our findings are consistent with earlier research suggesting that older adults who actively used ICT experience improved cognitive function, increased autonomy, and enhanced emotional outcomes (Czaja and Sharit 2009, Charness and Boot 2016).

The research also found that younger residents who regularly used ICT exhibited a notable sense of independence, competence, and empowerment in their daily lives. Residents with higher levels of technological proficiency not only utilized ICT tools for personal needs, such as communication and entertainment, but also often took on informal leadership roles by supporting their peers with technology. These experiences illustrate how ICT use can foster self-efficacy, autonomy, and peer collaboration among residents in nursing homes. Prior research supports this connection, showing that engagement with technology among older adults can enhance feelings of control and competence, which are key factors in psychological well-being and healthy aging (Bandura 1997, Schreurs *et al*. 2017). Moreover, the act of helping others with technology not only reinforces learning but also promotes meaningful social interactions and a sense of purpose (Quan-Haase *et al*. 2017). Such peer-led support may be particularly valuable in nursing homes, where formal ICT training is often lacking, further highlighting the potential of technologically skilled residents to support and empower their fellow community members.

### Barriers: limited resources, infrastructure, and literacy

Despite the recognized benefits of ICT, substantial barriers hinder its effective adoption, particularly in rural nursing homes. These barriers include limited Wi-Fi connectivity, the cost of devices, usability issues, and a lack of institutional support. Many residents face financial constraints that prevent them from owning personal devices, while others struggle with digital literacy and the complexity of technological interfaces. These difficulties are reflected in literature, where older adults, especially those in rural or underserved settings, often struggle with the complexity of technological interfaces and lack tailored training to support use (Heart and Kalderon 2013, Peek *et al*. 2014, Boamah *et al*. 2021a, 2021b, Li *et al*. 2025).

In addition to individual-level challenges, systemic limitations persist. Staff frequently lack the digital competence needed to support residents, underscoring the need for comprehensive training programs for both staff and residents in nursing home environments. Without institutional commitment to digital infrastructure and support, even motivated users may face barriers to ICT engagement (Charness and Boot 2009, Mitzner *et al*. 2010).

### Embedding findings into environmental gerontology

The research findings align closely with the principles of environmental gerontology, particularly Lawton’s (1982) P–E Fit model. This framework posits that older adults’ well-being depends on the alignment between their personal competencies—cognitive, physical, and emotional—and the opportunities, supports, and demands present in their environment. In our study, residents who had access to functioning equipment, demonstrated digital proficiency, and received institutional or peer support exhibited a high P–E fit. These participants were not merely using technology; they were actively shaping their digital environment to support social connection, cognitive engagement, and emotional well-being. This indicates that when the environment is structured to accommodate residents’ abilities, ICT becomes a tool for autonomy, self-efficacy, and meaningful participation in daily life. In contrast, residents with limited access to devices, unreliable internet, or insufficient guidance experienced low P–E fit. Their digital environment failed to meet their needs, resulting in exclusion from ICT use, restricted social interaction, and limited cognitive stimulation.

These observations extend the application of environmental gerontology into the digital domain, as suggested by Wahl and Gerstorf (2020), who argued that older adults were increasingly required to adapt to digital technology as part of their immediate environment. This also reflected Gitlin’s (2003) perspective that older adults actively modified their environments to meet their evolving needs. Their ability to adapt to digital tools, despite age-related challenges, demonstrated resilience and a proactive approach to maintaining quality of life. When the digital environment is inclusive, through accessible infrastructure, affordable devices, and available training, it has the potential to enhance well-being, promote autonomy, and support cognitive health for older adults in long-term care settings.

### Implications for policy

Policymakers play a critical role in reducing digital disparities between rural and urban long-term care facilities. Our findings suggest the need for targeted public investment in digital infrastructure within rural nursing homes, including funding for high-speed internet access, digital devices, and staff training. Moreover, policies that incentivize digital inclusion and promote equity in access to communication tools can help mitigate the social isolation that disproportionately affects older adults in rural areas. Federal and state agencies should consider integrating ICT implementation into broader public health and aging initiatives to ensure more inclusive and connected care environments.

### Limitations and future research suggestions

Despite applying all available resources to conduct this research, several limitations should be acknowledged. First, the sample size was relatively small. We reached out to five rural nursing homes, and only two agreed to participate. While we initially recruited 20 residents, only 16 were ultimately interviewed due to participants’ medical conditions. Furthermore, all 16 participants were White, reflecting the demographic makeup of southern Illinois. As such, the sample may not represent the broader population of nursing home residents across diverse regions. Second, some participants experienced speech or communication difficulties, which may have resulted in missed or incomplete responses to certain interview questions. These limitations suggest caution in generalizing the findings.

Future research should aim to include a larger and more diverse sample, encompassing multiple racial and ethnic groups, to better reflect the broader population in rural long-term care settings. Additionally, combining both qualitative and quantitative methodologies could offer a more comprehensive understanding of ICT’s role in enhancing the lives of nursing home residents.

## CONCLUSION

The findings of this study indicate that ICT use can enhance social connectivity, alleviate feelings of isolation, and contribute to an improved quality of life for residents in rural nursing homes. Successful engagement with these technologies, however, often depends on the availability of community support, assistance from staff, and residents’ access to reliable devices and internet connectivity. The results highlight the importance of considering both individual competencies and environmental supports when implementing ICT interventions in long-term care settings. Further investigation into strategies that optimize person–technology fit can provide valuable guidance for enhancing well-being among older adults in nursing homes.

## Data Availability

The datasets generated during and/or analyzed during the current study are available from the corresponding author on reasonable request.
